# Using a lottery to resolve indeterminacy when allocating resources for drugs for rare diseases

**DOI:** 10.1136/jme-2023-109723

**Published:** 2025-05-12

**Authors:** Kenneth Bond, Lars Sandman, Erik Gustavsson

**Affiliations:** 1 Department of Health, Medicine and Caring Sciences, Linköping University, Linköping, Sweden; 2 Department of Culture and Society (formerly Department of Culture and Communication), Linköping University, Linköping, Sweden

**Keywords:** resource allocation, ethics, decision making, philosophy, policy

## Abstract

Healthcare resource allocation decisions for high-cost drugs for rare diseases (DRDs) raise several challenges for decision makers, and, given the complexity of the decisions and the limited funding available for DRDs, it is reasonable to anticipate indeterminacy arising about which DRDs to fund. We argue that when indeterminacy does arise, one might consider resolving it by using a lottery. We examine the extent to which a lottery and the commonly used process of first come, first served satisfy the requirements of formal equality and several substantive and procedural values. We then examine two practical issues that arise when implementing a lottery, identifying the lottery participants and determining what happens to the ‘losers’ of a lottery, to examine the extent to which various ethical issues are raised by these practical decisions. We conclude that, while lotteries may not be used frequently to allocate healthcare resources, at least under the conditions outlined here, a random selection process has a morally justified role in allocating funding for DRDs and may sometimes be preferable to first come, first served.

## Introduction

 Healthcare systems globally strive to provide efficient and equitable care to their citizens. To support healthcare decision makers in meeting these goals, many countries are using health technology assessment (HTA) and deliberative decision processes in priority setting. Organisations using these evidence-based deliberative processes are frequently committed to the ‘accountability for reasonableness’ framework (A4R) developed by Daniels and Sabin’,^1 2^ or a similar framework, which requires that a rationale for a decision be publicly provided to ensure fair and legitimate healthcare resource allocation decisions.^3^ The basic idea of accountability for reasonableness is that, in the absence of an agreement on the substantive basis for a healthcare resource allocation decision, the fairness and legitimacy of the decision rests on a fair process, including ‘thorough deliberation about the facts, reasons and principles that are relevant to the dispute’.^3^ This commitment to substantive equality and procedural fairness may be enshrined in legislation—as in Sweden’s ethical platform for priority setting^4^—or embedded in the formal process documents of an HTA organisation, as part of its broader commitment to principles of transparency and accountability, as in the case of England’s National Institute for Health and Care Excellence.^2^ As part of these processes, decision makers appeal to broadly accepted ethical principles and processes, such as need, overall individual or population benefit, cost-effectiveness and equality, to decide how to distribute scarce healthcare resources.^5^ Nevertheless, despite this commitment to very similar substantive and procedural principles, healthcare resource allocation decisions across health systems will vary.^6^

Within this context, decisions about how best to allocate healthcare resources for the reimbursement of high-cost drugs for rare diseases (DRDs) raise several substantive challenges for decision makers. More specifically, uncertainties regarding the size of patient populations, the nature and course of rare conditions and high prices associated with DRDs can frequently lead to cost-effectiveness estimates well above what is considered cost-effective for drugs for more common conditions. While the definitions of rare diseases vary by jurisdiction, they are often characterised as conditions with a low prevalence, around 40–50 cases/100 000 people or fewer, and are also sometimes characterised as being conditions that are life threatening or severely debilitating.^7^ The frequent high cost of many DRDs^8^ and the uncertainty regarding their long-term effectiveness has led to vigorous debate about whether prioritising treatments for rare diseases over treatments for more prevalent conditions is morally justifiable.^4 9 10^ While DRDs may not necessarily require *higher priority* in resource allocation decisions, it might be argued that healthcare systems committed to justice and fairness owe patients *at least as great* an opportunity as other patients to receive promising treatments. Reflecting this duty on behalf of healthcare systems, HTA systems in many high-income countries have established separate or adapted reimbursement assessment and appraisal processes for newly licensed DRDs.^8^ These ‘supplemental processes’ are often justified by the fact that the application of standard assessment and appraisal criteria would lead to the frequent rejection of DRDs.^8^ Supplemental processes may accept less robust evidence of effectiveness or accept less cost-effective treatments for DRDs, and an argument for compensating in this latter way has been made on formal equality grounds.^4^ These egalitarian claims are also implicit in the willingness of some countries to dedicate funding for DRDs, for example, England’s Innovative Medicines Fund^11^ or Bulgaria’s dedicated budget for DRDs.^8^ Even with specific criteria for a DRD to be covered by these dedicated budgets, the number of drugs is likely to exceed the available funding. Given the limited funds, it seems inevitable that difficult decisions will need to be made about which promising new treatments should be publicly reimbursed.

Given the empirical and normative complexity of healthcare resource allocation decisions, situations may arise in which the principles and values guiding these decisions do not provide a decisive reason for who or what should receive priority.^12^ When faced with choices involving a duty to distribute a benefit fairly—yet the benefit is insufficient for all potential recipients and cannot be shared or divided equitably—there is a strong intuitive sense that fairness requires the use of an equal-chance procedure, such as a coin toss or lottery, to determine who should receive it.^13–16^ To account for the apparent strength of this intuition, some writers have contended that the use of a lottery constitutes a moral rule or principle.^16–18^ Using lotteries to resolve such situations in healthcare gained renewed attention during the COVID-19 pandemic, as decision makers wrestled with the question of how to fairly allocate intensive care unit (ICU) spaces, mechanical ventilators,^19^ drugs^20^ and vaccines.^21^ Recent empirical studies have indicated that public and professional decision makers and the general public view lotteries as an acceptable option for allocating healthcare resources,^22 23^ while the views of healthcare professionals vary depending on the allocation scenario.^24^

The aim of this article is to explore to what extent it is morally justified to use a lottery as a decision-making factor to resolve reimbursement decisions when other factors do not provide a decisive answer, and, given it is justified, to explore whether a lottery may have advantages over a process of first come, first served (FCFS). We begin by examining the conditions under which lotteries are generally accepted and argue that some reimbursement decisions for DRDs will satisfy those conditions. We then examine two different scenarios in resource allocation decisions for DRDs in which those conditions may be met, and assess the extent to which using a lottery in these scenarios reflects various formal, substantive and procedural values. Next, we examine two practical issues that arise when using lotteries in these scenarios, and the extent to which using a lottery may have advantages over FCFS. We conclude by summarising the results of our analysis and noting some remaining uncertainties and challenges. To emphasise, our concern here is with the public reimbursement of DRDs and patient access to publicly funded treatments, which are distinct from decisions regarding drug development and market licensing.

## The case for lotteries for drugs for rare diseases

The strongest justification for a lottery requires that three criteria be satisfied: (1) there must be scarcity of a good such that it cannot be distributed among all potential recipients; (2) there is indeterminacy regarding how to distribute the good in question based on substantive principles and (3) fairness or procedural justice requires that a recipient of the good be selected impartially.^13–16^ While scarcity and indeterminacy jointly make the use of a lottery morally permissible—but not obligatory, as one might simply ‘pick’ an option in such cases—a commitment to procedural fairness makes a lottery (and possibly other similar selection procedures) morally required.^15 25^ The requirement for a lottery is based on the fact that if morally relevant reasons cannot be given for selecting one recipient over another, impartiality demands that a process must be used that protects one recipient being selected over the other for morally irrelevant reasons.^[i]^ Lotteries, it is argued, remove reasons altogether, so are uniquely able to maintain impartiality when selecting recipients.^25 26^

Drugs (including DRDs) can enter publicly funded healthcare benefits packages in two main ways, each of which reflects a different relationship between healthcare decision makers and budget holders. In one scenario, a national body may have control over both the reimbursement decision and the budget for the DRD, as is the case with England’s Innovative Medicines Fund. Another scenario—found, for example, in Sweden—involves a national body that recommends which drugs ought to be funded, while the actual budget responsibility lies with individual care providers (such as hospitals and clinics), who may find themselves unable to follow these recommendations for all patients due to budget constraints.^[ii]^ As we will argue below, there are good reasons to believe that the three conditions making a lottery morally required may sometimes apply in these scenarios.

### Scarcity

Lotteries in healthcare are typically proposed when the supply of a good is limited either by nature (eg, organs) or by its manufacture and distribution (eg, vaccines). When a good is scarce, it cannot be divided without losing the primary characteristic for which it is valued (often referred to as ‘indivisibility’).^15^ This is true for DRDs, which have a minimally effective dosing regimen needed to achieve their expected benefits. Decisions to fund DRDs involve healthcare goods that are usually limited not by nature, but by the means (money) with which to procure the good. While it can be difficult to persuade people that healthcare budgets cannot be expanded infinitely to accommodate new treatments,^27^ limited budgets are real constraints on healthcare decisions. This constraint has been graphically illustrated using a bookshelf metaphor, which shows that, under resource limits, difficult decisions must be made regarding how effective a health technology needs to be for it to be considered ‘effective enough’ for inclusion in a healthcare benefits package, as well as the trade-offs in maximising population health that may be necessary to account for other ethical values.^28^

### Indeterminacy

When one of two DRDs can be selected because there are stronger reasons for it being selected based on substantive moral principles or values (eg, severity of condition treated, overall benefit, cost-effectiveness), the choice between the alternatives is *determinate*. When substantive principles do not provide a decisive reason for allocating resources to one DRD over another, the choice is *indeterminate*.^25 29 30 [iii]^ Indeterminacy can arise in several ways: two options may be equally good; the decision-maker may lack sufficient information to determine whether one option is better or they may be unable to rank the options at all.^25^ Indeterminacy of the first kind is observed in microallocation decisions in healthcare, for example, when allocating patients to ICU spaces or other urgent treatments.^31^ In these cases, the relevant moral considerations are judged to be equal*,* and the lottery is a ‘tie breaker’.^32 33^

Tie-breaking situations are unlikely to occur when making decisions about DRDs. However, indeterminacy based on lack of information is an acknowledged challenge, both at the policy level and at the clinical level. Many DRDs are introduced into healthcare systems with smaller studies using weaker study designs, shorter studies even if the drug is supposed to benefit the patient for a long time (eg, gene therapies with an expected effect that is life-long) and surrogate outcomes that need to be translated into clinical effects.^8^ This is also true for individual patients, when it comes to DRDs. Even if there are potential differences in how a drug will benefit particular patients, this is often impossible to predict at the clinical level, and the effect of treatment is not known until a patient has received treatment.

When it concerns the third type of indeterminacy, numerous ‘value frameworks’ have been developed to help policy makers assess the relative value of healthcare technologies (though few specifically designed for DRDs).^5^ The complexity of these frameworks reflects the fact that priority setting involves a plurality of normative judgements, each of which may be given a different weight in different contexts.^23^ While these frameworks may have a common core of attributes such as health benefit, affordability and societal impact,^5^ there is a tendency to add ‘supplemental’ or ‘novel’ considerations,^34^ such as accepting greater uncertainty in clinical evidence and economic evaluation, expert input regarding potential treatment benefit and consideration of ‘unmet need’,^8^ to more fully account for what seems to matter in decisions regarding DRDs. These additional considerations further complicate the moral pluralism already inherent in standard frameworks. Furthermore, these frameworks seldom give any more detailed guidance about how different values should be balanced against each other, and there might be different interpretations of how the factors should be interpreted and whether they actually apply, and if so, to what degree.^35^ As a consequence, indeterminacy in decisions regarding which DRDs to fund may arise from ignorance of empirical facts, a lack of clarity regarding normative considerations or a combination of both.^36^ Being in a position of not knowing does not necessarily imply that it is impossible to know,^36^ although that may be the situation. For example, depending on one’s view of the nature of moral knowledge, there may be a fact of the matter about which DRD is better, but the fact may remain inaccessible to us.

Methods of HTA, and resource allocation decisions more broadly, typically assume that a treatment can be related to an alternative in one of three ways: it may be better than, worse than or equal to the alternative.^29^ While ignorance provides a plausible explanation for indeterminacy in the context of resource allocation decisions, there is another possible source of indeterminacy: alternatives may be ‘on a par’.^29^ Alternatives are said to be ‘on a par’ when (1) one alternative is better in some relevant respects, (2) the other is better in other relevant respects and (3) neither appears to be at least as good as the other when all things are considered.^29^ Herlitz^12^ has argued that the need to consider more than one overarching value in healthcare priority setting, for example, both health maximisation and allocation according to need, will plausibly (and perhaps not infrequently) produce parity among options in high-level healthcare resource allocation. In the case of public funding for DRDs, parity may also plausibly arise. One may face a choice between two DRDs, one of which targets a severe condition in adults, but with greater uncertainty about its effect, and, thus, about its cost-effectiveness, although it will be relatively easily implemented. In comparison, the other DRD targets a somewhat less severe condition in children but with somewhat greater certainty regarding its effect or better cost-effectiveness, although it has some anticipated challenges in implementation, such as limited availability of diagnostic testing.

### Just healthcare distribution and fair process

As noted above, lotteries have been proposed to resolve indeterminacy when there are no substantive reasons to select one patient over another when allocating scarce healthcare resources. When substantive criteria are unable to determine which of two patients should receive a treatment, the lottery is seen as a way to select a recipient fairly. A lottery accounts for fairness by ensuring the indeterminacy is resolved impartially and is not ‘distorted by some form of unequal concern’.^17^ As noted in the ‘Introduction’ section, healthcare resource allocation decision processes embody decision makers’ commitment to procedural justice. The justice-based requirement to provide principled reasons for resource allocation decisions requires that, when relevant moral reasons are unavailable, morally irrelevant reasons—such as sexism, ageism or conflicts of interest on the part of decision-makers—must also be excluded from the decision-making process.

If the patients, their conditions and the treatment benefits and costs were sufficiently similar, it might be tempting to frame the comparison between two drugs as a question of which of two groups—differing in size—ought to be helped. However, the action of a drug is not dichotomous (working or not) and a comparison will consider many differences such as condition severity, likelihood of benefit, cost, different thresholds for cost-effectiveness. Because of this, it is possible that when two DRDs are compared, one drug might be more effective than the other and treat a slightly more severe condition yet have worse cost-effectiveness and lower budget impact (because it treats fewer patients).

In summary, the conditions that require the use of a lottery (scarcity of the resource, indeterminacy with respect to who should receive the resource and the need for a process that accounts for procedural fairness among potential recipients) can be present when allocating funding to DRDs at both the level of allocating a limited budget to specific DRDs and when allocating a budget to fund a DRD for a defined group of patients. In the first scenario, macro-level reimbursement decisions have used substantive criteria to determine clear cases of DRDs that warrant funding and ones that do not. However, there are likely to be cases in which it is indeterminate whether an eligible DRD should be funded or not in relation to the criteria. These indeterminate cases constitute a group of DRDs some, but not all, of which could be funded within the remaining budget and a ‘drug lottery’ could be used to resolve the indeterminacy regarding which ones to fund. This situation might be encountered in countries with national funds that are ring-fenced for innovative drugs that do not meet common thresholds for cost-effectiveness, for example, England and Bulgaria.^8^ In the second scenario, a national body has recommended the use of a DRD for eligible patients; however, the responsibility for funding lies with the healthcare region or hospital clinic, and the budget is insufficient to provide the DRD to all eligible patients. This situation might occur in Sweden, for example, where there are national processes for recommending new drugs with the aim of ensuring national equality, but regional councils (or county councils) are responsible for funding primary and secondary care.^37^ While the eligibility and priority of many patients may be determined using substantive criteria, there will be cases in which the substantive criteria do not fully determine priority among patients and a ‘patient lottery’ could be used to resolve the indeterminacy. Because the higher-level reimbursement recommendation has been made based on substantive principles, there is no justification for withholding the treatment from all patients; it ought to be provided to at least some.

## Decisions, lotteries and values

When there is indeterminacy regarding who should receive a good, lotteries may account for formal, procedural and substantive values.^17^ Procedural values are related to how a decision-making process is conducted and include values such as impartiality, transparency and accountability.^3 38^ Substantive values are the basis for the normative criteria on which resource allocation decisions regarding DRDs will be made and typically include values such as need, capacity to benefit, and cost-effectiveness.^8^

It has been argued that the chance provided by a lottery provides a ‘probabilistic division of the good *ex ante*’^14^ or the ‘surrogate satisfaction’ of claims to the benefit being provided.^13^ There are several difficulties with such arguments, chief among them are how to convincingly explain how a chance can function as a proxy for receiving the good itself^17^ and how to avoid counterintuitive implications of this view—such as the idea that one’s claim to a good is satisfied despite losing the lottery.^39^ Hence, we will ignore this account of lotteries in the subsequent analysis.

The strongest argument for a lottery is that it is required to resolve indeterminacy because of a justice-based claim for procedural fairness.^15 25 26^ As described above, this commitment to procedural fairness is consistent with the focus on procedural justice in healthcare resource allocation decision processes,^1 2 38^ and which has influenced HTA processes globally. Since lotteries account for several substantive and procedural values, this commitment to formal, substantive and procedural values provides a justification for using a lottery to resolve indeterminacy in selecting DRDs for reimbursement (a drug lottery) and between patients to receive DRDs (a patient lottery).

Lotteries may also account for values such as the individual and social utility gained or lost by the knowledge that one may have a chance of receiving or not receiving a potentially effective treatment.^40^ The result of a lottery may also be emotionally easier for decision-makers and those not selected to accept than the outcome of some other decision-making process.^13^ While these two considerations are important, we focus on the values for which a stronger case has been made to justify using a lottery.

### Formal values

Formal equality, as it is commonly formulated, implies that cases that are equal in morally relevant ways should be treated equally and cases that are unequal in morally relevant respects should be treated unequally.^4^ Formal equality is a reminder that, if the morally relevant features of a situation do not give us reason to give one potential recipient priority over the other, then no other feature of the situation should be used to do so because that would treat them unequally arbitrarily. By using a mechanism of chance rather than a possibly morally irrelevant feature of the potential recipients, a lottery accounts for formal equality.

### Substantive values

We can think of equal treatment in two ways: either as providing the same *treatment* to meet the same medical need, or as giving equal *consideration* to individuals’ needs when deciding who should receive treatment. By offering equal treatment in the second sense, we are obligated to provide equal treatment in the first sense, if possible. When we are unable to provide equal treatment in the sense of offering the same therapy, fairness requires that we give each potential recipient an equal chance of receiving the good.^41^ This ability to demonstrate a positive commitment on the part of the decision-maker to give equal consideration to the relevant substantive factors is referred to as the ‘expressive function’ of a lottery.^15^ The value of equal chances reflects the importance we attach to people’s prospects (prospect-regarding equality) of achieving a desirable outcome, in this case, the prospect of receiving therapy that may improve their health.

### Procedural values

Impartiality is an important procedural value of fair resource allocation decisions. Impartiality refers to a decision process, and those involved in it, being free from undue influences,^1^ either positively or negatively. By using a chance mechanism to select recipients, lotteries rule out any problematic reasons such as bias or partiality^15 25 26^—this ability is referred to as the ‘prophylactic function’ of a lottery.^15 [iv]^

Two other procedural values, transparency and accountability, support impartiality. Despite the obvious strength of the lottery with respect to impartiality, people may have different intuitions about whether a lottery or some other process, such as FCFS, better accounts for transparency and accountability. Transparency demands that the criteria and process by which a decision is made are described and can be understood by those affected by the decision and by any interested party.^1^ Those subject to any selection process should have some assurance that it has been properly employed and had appropriate oversight. When a lottery is required, a principled process is used to select lottery participants, so complete transparency is possible up to the point of the random mechanism selecting a ‘winner’. Many people are familiar with random mechanisms used in other contexts, so the process of being selected by chance is not mysterious (although it involves second-order rather than first-order reasons for the selection).^25^

Accountability, in the context of priority setting, means being able to justify resource allocation decisions by giving the reasons for why a treatment or patient has been given priority.^1^ In the case of a lottery, we can explain the principled selection process that produces the pool of lottery participants and how one candidate was selected over another, but because the final allocation is made randomly, we might consider this partial accountability rather than complete accountability.

### Does lottery placement matter?

Do the drug lottery and patient lottery differ morally? To answer this question, we must examine whether the two lotteries differ with respect to the extent to which they account for the substantive and procedural values just described. The decision to use a drug lottery or a patient lottery is motivated by indeterminacy and the need to account for procedural justice at different levels of decision making. Regardless of the source of the indeterminacy (equality, ignorance or parity), a lottery accounts for substantive equality and various procedural values (impartiality, transparency and accountability). Because the conditions for a lottery have been met in both scenarios, whether the indeterminacy is between two DRDs for two different conditions or between two individuals with the same condition, the values described above are equally well accounted for. One might think that the risk of violating formal equality is arguably somewhat less with a drug lottery than with a patient lottery because the members of a group who have the same condition and need for treatment are more similar to one another in all relevant respects (patient lottery) than are members of two groups with different conditions and treatments (drug lottery). However, in both cases, we have reached a point of indeterminacy with respect to which drug or which patient should be given priority based on substantive criteria, so there is no difference in terms of formal equality.

In summary, both lotteries will account for the formal, substantive and procedural values described above. Nevertheless, a drug lottery appears somewhat fairer than a patient lottery because individuals with the same condition are being treated similarly. This is important because, as we know from other contexts, having access to a life-changing treatment can alter one’s identity as a person with particular prospects. It is reasonable to think that this identification may be stronger if I know my patient group has access to a DRD than if it does not. However, to what extent this is the case seems to be an empirical question.^42^

Are there other factors that make the use of a lottery more acceptable in one scenario rather than the other? There may be psychological effects that make the lottery more acceptable in a given scenario. For example, patients who lose a drug lottery may feel more fairly treated than those who lose a patient lottery because they know that all people like them receive or do not receive the drug. That said, a patient lottery may be easier for patients and healthcare professionals to accept because the use of a lottery to determine allocation among individuals is more familiar due to its use during the COVID-19 pandemic (a kind of availability bias).

### Lotteries versus first come, first served

We now consider how a drug lottery and a patient lottery might function in practice, examining the ethical issues their implementation may raise and how they compare with an alternative selection method, FCFS. Typically, new drugs are assessed by regulatory bodies in sequence and, partly as a consequence of this, they are also evaluated and appraised consecutively for reimbursement. For this reason, reimbursement decisions are made typically using the process of FCFS. Clinical settings also employ FCFS. When too little is known about patients to prioritise them according to substantive criteria or when patients’ claims are considered to be equally strong, FCFS is used to determine which patients will receive treatment next.^18^

To describe in more detail how the lotteries may be used, in our reimbursement scenarios neither the drug lottery nor the patient lottery is the primary or even a frequently used process for determining funding; lotteries are employed to resolve indeterminacy. To be eligible for funding, information about the condition and DRD will have to show that the treatment of the condition meets substantive reimbursement criteria. During a budget year, a number of the new DRDs will clearly satisfy these criteria and be reimbursed, and some will clearly not satisfy these criteria and not be funded. In addition, a number of drugs will be indeterminate cases in relation to the criteria and, therefore, likely to also be indeterminate in relation to each other. Hence, even if there are funds left at the end of the year, and some more drugs could be funded, it will be unclear which DRDs should receive this funding. In this situation, a drug lottery can be used to resolve the indeterminacy.

In the case of a patient lottery, a national HTA or similar body provides a recommendation to the health region (and to healthcare institutions) to fund a DRD for eligible patients and it is left up to the regions and the hospitals and clinics to manage their budgets to provide access. However, the cost of treating all eligible patients within the hospital’s or clinic’s care might be larger than the available funding. Hence, the hospital or clinic will have to choose which patients will receive the DRD and which will not. There might be patients who are likely to benefit more from the drug, or who have a worse condition, and who therefore should be prioritised. There will also be patients who are likely not to benefit or who are not as severely ill. However, in at least some cases, it might be difficult to distinguish between different patients and the care provider needs some way to decide who should get access to the drug and who should not. In this situation, a patient lottery can be used to resolve the indeterminacy.

It may appear reasonable to employ FCFS to determine funding and to use a lottery when time cannot be used as an identifying factor for some reason. Nevertheless, even if FCFS is a possible option, as we will argue below, there may be reasons why one should use a lottery. Choosing to use a lottery over FCFS requires showing that a lottery may be at least as good as FCFS, morally speaking, and is better in some other important respect.

### Accounting for impartiality

FCFS is believed to account for the procedural and substantive values described in the previous section to the extent it can secure *impartiality* and *equiprobability*.^43^ In addition, by making use of a natural process (queuing behaviour),^43^ FCFS is thought to avoid the cost of administration and coordination required to pool recipients for a lottery, thus better respecting the value of using the fewest system resources in terms of time, human resources, infrastructure and administrative effort. However, each of these putative advantages is open to question.

Proponents of FCFS believe that it accounts for impartiality because it is assumed that there is no general correlation between individuals (or drugs) and their position in a queue.^43^ By having potential recipients follow the same queuing procedure, the decision maker expresses a commitment to procedural equality.^43^ Views are mixed as to whether the position in a queue created by FCFS is sufficiently independent of the characteristics of the potential beneficiaries for places to be considered unbiased. Some healthcare ethicists have concluded that an equal chances lottery is more justifiable than FCFS because a lottery is less likely to be corrupted by participants.^18^ Surveys of the general public have found FCFS to be perceived as fairer than a lottery for allocating scarce healthcare resources, perhaps because FCFS is perceived as a proxy for allocating resources according to need^22 23 [v]^ or because it accounts for waiting time.^44^

As noted above, the values of transparency and accountability support impartiality, and a lottery appears to perform better than FCFS with respect to both. FCFS is arguably less transparent than a lottery because it is more difficult to describe how drugs or people get to the front of a queue. While FCFS does use a principled process up to the point at which recipients enter the queue, the only reason that can be given for the recipient being at the front of the queue is that they got there first. There may be factors involved in FCFS that do not justify choosing the first in line, for example, when someone is given the opportunity to jump the queue and gain a more favourable position.

In using FCFS in place of a drug lottery, one might be concerned that the ability to secure a position in the funding queue results from complex considerations and processes such that allocating funding according to FCFS will disadvantage a drug based on a company’s time for drug development.^44^ Factors influencing development time include decisions about which condition to target—often shaped by advocacy groups and other stakeholders—as well as the complexity of the condition and the size of the relevant patient group. These elements affect the size and complexity of the clinical trials, which in turn influence the speed and conditions of regulatory approval, ultimately determining how and when the drug is assessed by an HTA body.

In using FCFS in place of a patient lottery, it has been argued that FCFS cannot prevent morally irrelevant factors, such as wealth, social position or having access to certain services (transportation, child care), from influencing who gets to the front of the queue.^18^ In addition, since people must proceed through diagnostic procedures to get a place in line, using FCFS to resolve indeterminacy among patients assumes that people are materially equal and that diagnostic and prognostic decisions are not linked to factors outside of the healthcare system (ie, to determinants of health).^45^ Opponents of FCFS argue that both assumptions are unrealistic.

In defence of FCFS, one might argue that addressing the range of potential inequities that influence when and how drugs are developed and assessed, and when and how patients seek care, goes beyond what we should expect from a resource allocation process. While resource allocation decisions are means to ensure fairness in allocating scarce resources, we should not expect or design our resource allocation decisions to remedy inequities that result from institutions and processes outside of healthcare. Nevertheless, even those who defend the ability for FCFS to account for impartiality and equiprobability in principle have conceded that, in practice, ensuring the fairness of FCFS may require ‘careful oversight and levelling policies to help ensure that social privilege does not confer better chances.’^43^ While this concession refers to *known* factors, our concern, stated above, also includes protection from *unknown* factors and *structural* advantages that may confer benefits without requiring awareness on the part of those privileged by them. Furthermore, the need for careful oversight and additional policies may mean that the administrative and resource advantages ascribed to FCFS are not realised in practice—or at least not to the extent commonly assumed.

What about equal chances? Proponents of FCFS formulate equal chances in terms of epistemic equiprobability: so long as the decision-maker (or lottery participant) has no evidence that one drug or person is more likely than another to be first in line, their ‘rational degree of credence’ in each drug or person getting to the line first is equal and, therefore, epistemically equiprobable.^43^ In formulating equiprobability this way, the argument for FCFS appears to take the form of an argument from ignorance. However, as in other areas of medicine and healthcare, when moral stakes are high, the equiprobability of the decision procedure (and the fairness it confers to all potential beneficiaries) is preserved only by excluding the influence of both *known* background factors and *unknown* factors—including the myriad and complex factors involved in social and political environments. Only a statistically (or objectively) equiprobable process, such as a lottery, can achieve this.

Relatedly, to account fully for the procedural values of impartiality and transparency, it seems that the decision-maker and those affected by the resulting allocation decision believe that the selection process provides equiprobability. Transparency refers to the ability to trace the workings of the lottery payoff conditions and make reasonably accurate predictions of the probabilities that attach to various outcomes. While an allocator using FCFS may be able to calculate non-fractional probabilities in principle,^43^ in practice, the process is opaque.^14^ However, it is important to acknowledge that, unlike a lottery, FCFS accounts for waiting time and will be fairer in that respect. Nevertheless, we ought not to allow impartiality and fairness to be contingent on our ignorance when a more robust method for securing fairness, namely, statistical equiprobability, is available.

If we ought to use a lottery rather than FCFS in cases where potential recipients continue to enter over time—as in both drug and patient lotteries—and where prognoses worsen as time passes, as is often the case with many rare diseases, then assembling a pool of participants involves identifying an ethically acceptable trade-off between procedural and substantive considerations.^40^ This trade-off requires waiting to allow more potential recipients a chance to benefit, even though doing so may lead to some deterioration in individual prognoses. Recall that in a drug lottery, the lottery is being used only when substantive criteria cannot make a determinate choice among DRDs. For this reason, the number of drugs that may be eligible for a lottery will likely be low and the lottery might take place once a year when all indeterminate cases have been identified.

In the case of a patient lottery, the lottery is employed when substantive criteria cannot determine priority among patients eligible to receive a DRD. In such cases, a lottery offers a procedurally fair method of selection when no morally relevant differences exist among patients. If FCFS were used instead, patients might be selected based on the time of referral or clinic registration. Consider a scenario where 20 patients qualify for treatment with a DRD, but funding is available for only 15. Ten of the patients are clearly prioritised to receive the drug, leaving indeterminacy among the remaining 10. The next five patients could be selected based on FCFS, but what justifies this order? If their level of need has already been accounted for, using their position in line becomes arbitrary and potentially unfair, for all the reasons discussed earlier. Instead, a patient lottery should be used to select the next five recipients. More complex scenarios could be constructed and exactly how the lottery is employed, and whether it is fair to use FCFS, will depend on complex factors. Nevertheless, we suspect in many cases a lottery may be more reasonable than FCFS.

Finally, manufacturers may prefer a patient lottery because it ensures a drug receives a positive public funding decision and the spending is controlled by prioritising patients. Patients may prefer a drug lottery because, once the DRD is approved for reimbursement, they will be assured access to the DRD rather than merely having a chance to receive it.

### What happens to the ‘losers’?

While we believe we have presented a plausible case for using a lottery to allocate resources for DRDs, challenges remain. A pressing concern arises in the context of iterative lotteries: should the ‘loser’ of a lottery—whether a drug in a drug lottery or a patient in a patient lottery—be eligible to participate in a subsequent round. The answer depends on one’s view of how considerations of procedural and substantive justice and principles of fairness in distributing particular goods are satisfied by a lottery. On the account of lotteries we have put forward, having a chance satisfies a commitment to procedural fairness. Recall that the ‘losers’ were eligible for funding or treatment based on substantive criteria, but there was indeterminacy compared with a second drug or patient. In light of this, it seems that losers should be eligible to participate in the lottery in the subsequent funding cycle. However, an iterative lottery may create unfairness of two kinds. First, if there are more participants in subsequent rounds than in previous rounds, the losers may consider it unfair that their chance of receiving funding or the drug in the new lottery is less than in the previous one. In contrast, under an FCFS system, those who do not receive the treatment or funding in one cycle move forward in the queue and, assuming steady funding, will eventually gain access. The difference between the two is that, with FCFS, patients can be certain that, at some point in time, they will receive funding or treatment (although it may be too late for treatment to be effective). This is not the case with a lottery, where there remains the possibility that an individual may never receive funding or treatment. To the extent that the cost of waiting should be taken into account, FCFS does so, whereas a lottery does not.^43^

A second challenge concerns the possibility of appeal. Appeal is considered a fundamental procedural principle of fair and legitimate healthcare resource allocation decision making,^3^ and its importance within reimbursement processes raises the question of whether a negative lottery outcome can be appealed. Under the terms of many reimbursement decision processes, when a negative recommendation or decision has been made, there is an opportunity for an applicant to appeal the decision (1) on procedural grounds, when it is believed the process was not properly followed or (2) on substantive grounds, when it is believed the recommendation or decision is unreasonable in the light of the evidence submitted^46^ or the relevant ethical values weighed by the recommendation body.

Losing a drug lottery is equivalent to the drug receiving a negative reimbursement decision (ie, no funding). When a lottery is used, an appeal could be made on procedural grounds, if the potential recipient had a reason to believe that the assessment and appraisal process up to and including the lottery had not been conducted according to the defined process (eg, as described in the HTA body’s process documentation). An appeal on substantive grounds challenges the reasons—whether based on empirical evidence or the weighing of ethical values—underlying a decision not to reimburse a drug or to impose limitations on the clinical indication for which it was approved. In the case of the lottery, the appeal would contest the claim that the reasons were indeterminate and argue that there was a determinate choice. For this to occur, there needs to be sufficient transparency in the process for the appellant to know the other eligible product. This degree of transparency may be difficult to achieve given the confidential nature of reimbursement processes and of the clinical and economic information that is used in reimbursement decision-making.

In a patient lottery, the drug has been recommended for reimbursement, and the indeterminacy is with respect to which of two eligible individuals should receive it. We are not aware of any health systems that allow individual clinical decisions of this kind to be formally contested, and it seems unlikely that appeals would arise in this context. While patients may object to not receiving treatment, many healthcare systems lack formal mechanisms for appeal at this level, particularly where there is no legislated right to healthcare. The situation may be different, however, in systems where such a right is established in law.

## Conclusion

There will always be tremendous pressure to ensure the cost-effectiveness of healthcare, and the biomedical research industry will continue to provide a steady and increasing stream of DRDs to market. As a result, indeterminacy will likely arise among options and there will be hard choices regarding which DRDs to fund. We have argued that lotteries provide a way to help resolve indeterminacy in resource allocation decisions for DRDs while accounting for important substantive and procedural values that are fundamental to legitimate and responsible healthcare resource allocation decisions. Furthermore, we have argued that there are good reasons why a decision maker may choose to use a lottery rather than FCFS. As others have noted, if lotteries are to be used, we will need to better understand how to implement them in a way that is minimally morally straining for healthcare workers and acceptable to the public.^33^

The frequency of indeterminacy, and especially parity, arising in decisions remains an interesting and important empirical question. Regardless of the actual frequency of cases, the validity of our argument and the application of our results in practice do not depend on there being a large number of indeterminate cases, and we think it is unlikely that there will be many. In any case, our argument concerns the principled question about the justification for and use of a lottery rather than the frequency of its use.

We have outlined two ways in which indeterminacy may plausibly arise in reimbursement decisions for DRDs. When such indeterminacy occurs, decision-makers have two primary options for resolving it: a lottery and FCFS. We have argued that a commitment to procedural fairness—grounded in the values of impartiality, transparency and accountability—favours the use of a lottery in certain circumstances. While lotteries may not have a prominent role in addressing DRDs, they are not widely used in healthcare generally. Nevertheless, if we commit to making our resource allocation trade-offs explicit, there may be a greater role for the lotteries in allocation decisions than is typically acknowledged. If the use of a lottery to resolve indeterminacy in funding or allocating DRDs is found to be objectionable, the burden lies with critics to demonstrate how reimbursement decisions can be made in a morally defensible manner—without presuming that healthcare budgets are infinitely expandable.

## Data Availability

No data are available.

## Footnotes

[i]We set aside the issue that the need to select a payoff condition for a lottery requires ‘picking’.

[ii]We expand on these scenarios in a later section.

[iii]Our interest in this paper is to determine to what extent a lottery might be justified in the clearest cases of indeterminacy, so we do not consider weighted lotteries.

[iv]It has been argued that a process of ‘first come, first served’ (FCFS) also satisfies the requirements of procedural fairness. We compare the use of lotteries and FCFS in the next section.

[v]While this may be the perception, the use of assessments of need in processes that employ FCFS, for example, in emergency departments, shows that place in queue does not seem to be a proxy for need.
